# Fusobacterium necrophorum Bacteremia With Evidence of Cavitary Pulmonary Lesion

**DOI:** 10.7759/cureus.19537

**Published:** 2021-11-13

**Authors:** Zain Mohiuddin, Taylor Manes, Andrew Emerson

**Affiliations:** 1 Internal Medicine, Western Reserve Hospital, Cuyahoga Falls, USA; 2 Internal Medicine, A.T. Still University - Kirksville College of Osteopathic Medicine, Kirksville, USA; 3 Family Medicine, Western Reserve Hospital, Cuyahoga Falls, USA

**Keywords:** pulmonary nodule, tonsilitis, lemierre’s syndrome, cavitation, internal jugular vein thrombophlebitis, pharyngitis, dysphagia, fusobacterium necrophorum

## Abstract

*Fusobacterium necrophorum (F. necrophorum) *is a gram-negative anaerobic bacterium and a known etiologic agent in Lemierre’s syndrome. This rare disease commonly presents with persistent sore throat and dysphagia, which can spread to involve the internal jugular vein. Presented in this report is an interesting case of a patient who presented with a progressively worsening sore throat, dysphagia, and productive cough on admission. Blood cultures were positive for *F. necrophorum *and computed tomography angiogram (CTA) of the chest detected cavitation in the left lower lobe and a large consolidation within the right lower lobe without evidence of a vascular defect. CT of the neck with IV contrast demonstrated no findings of abnormal vascular structures. This patient was diagnosed with pneumonia secondary to *F. necrophorum *bacteremia and treated successfully with antibiotics and was discharged home. Clinical suspicion is warranted in patients with worsening symptoms of sore throat and dysphagia, as this rare syndrome may be present.

## Introduction

*Fusobacterium necrophorum (F. necrophorum), *a gram-negative anaerobic bacterium, is the etiologic agent in 80% of Lemierre’s syndrome cases [1]. It is primarily considered part of the normal mouth flora, GI tract, and female genitourinary tract [2]. *F. necrophorum* is also commonly found with other mouth flora in dental abscesses, peritonsillar abscesses, and may be involved in pharyngotonsillitis [3]. When infectious, *F. necrophorum* commonly presents similar to *Streptococcus pyogenes* (Group A Streptococcal [GAS]) with a sore throat as the initial symptom. Typically, clinicians order a rapid test and throat culture for GAS, but *F. necrophorum* is difficult to diagnose from a throat swab, requiring an anaerobic culture on a selective medium, and currently, there is neither a rapid nor a PCR test available for use [1]. This organism is most commonly associated with Lemierre’s syndrome, which requires radiographic evidence of a thrombus in the internal jugular vein and a positive culture growing* F. necrophorum* or another pathogen for diagnosis [4]. This syndrome can lead to severe complications, including septic suppurative thrombophlebitis of the internal jugular vein and pulmonary septic emboli [2]. However, there is evidence of other clinical manifestations: cavitary lung lesions, recurrent tonsillitis, chronic purulent otitis, septic arthritis of the knee, and cerebral abscesses [5]. Therefore, clinical suspicion is warranted in patients with worsening symptoms of a sore throat to rule out this severe infectious condition, revealing a mortality rate of 5% [6]. Here, we describe an incidence of a cavitary pulmonary lesion complicated by *F. necrophorum* bacteremia in a previously healthy adult.

## Case presentation

**Figure 1:**
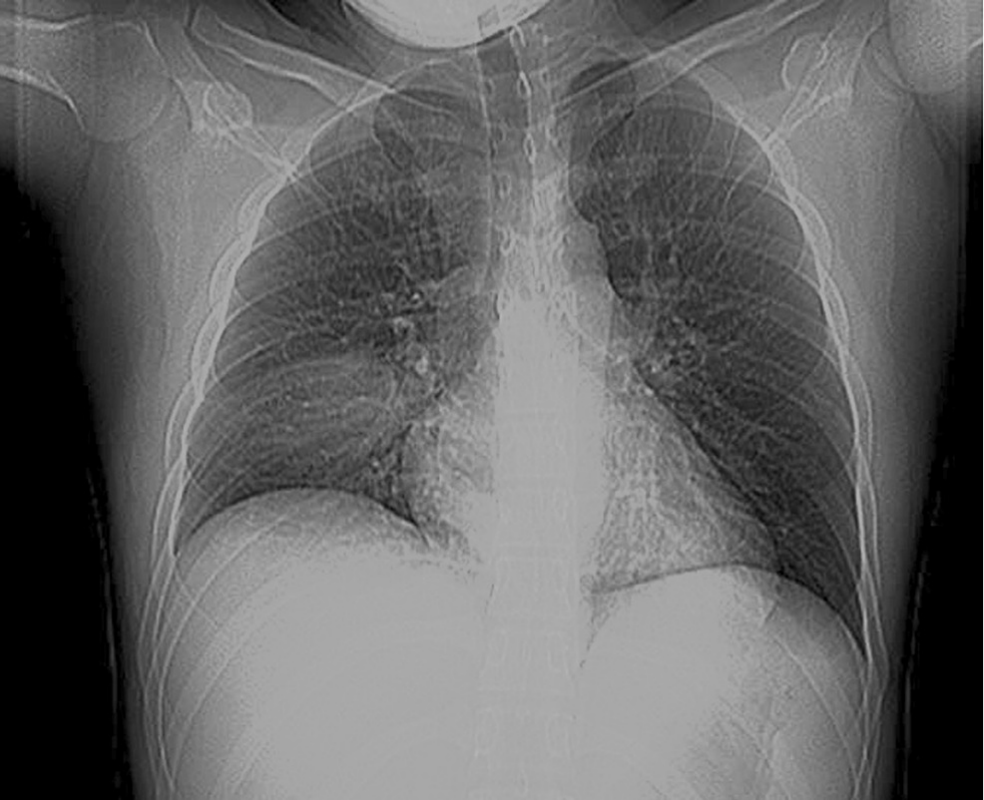
Portable X-ray of the chest. Portable chest X-Ray with an anteroposterior view demonstrating a consolidation in the right midlung, likely in the inferior right upper lobe. In this view, the left lung appears clear, likely due to the positioning of the patient.

**Figure 2:**
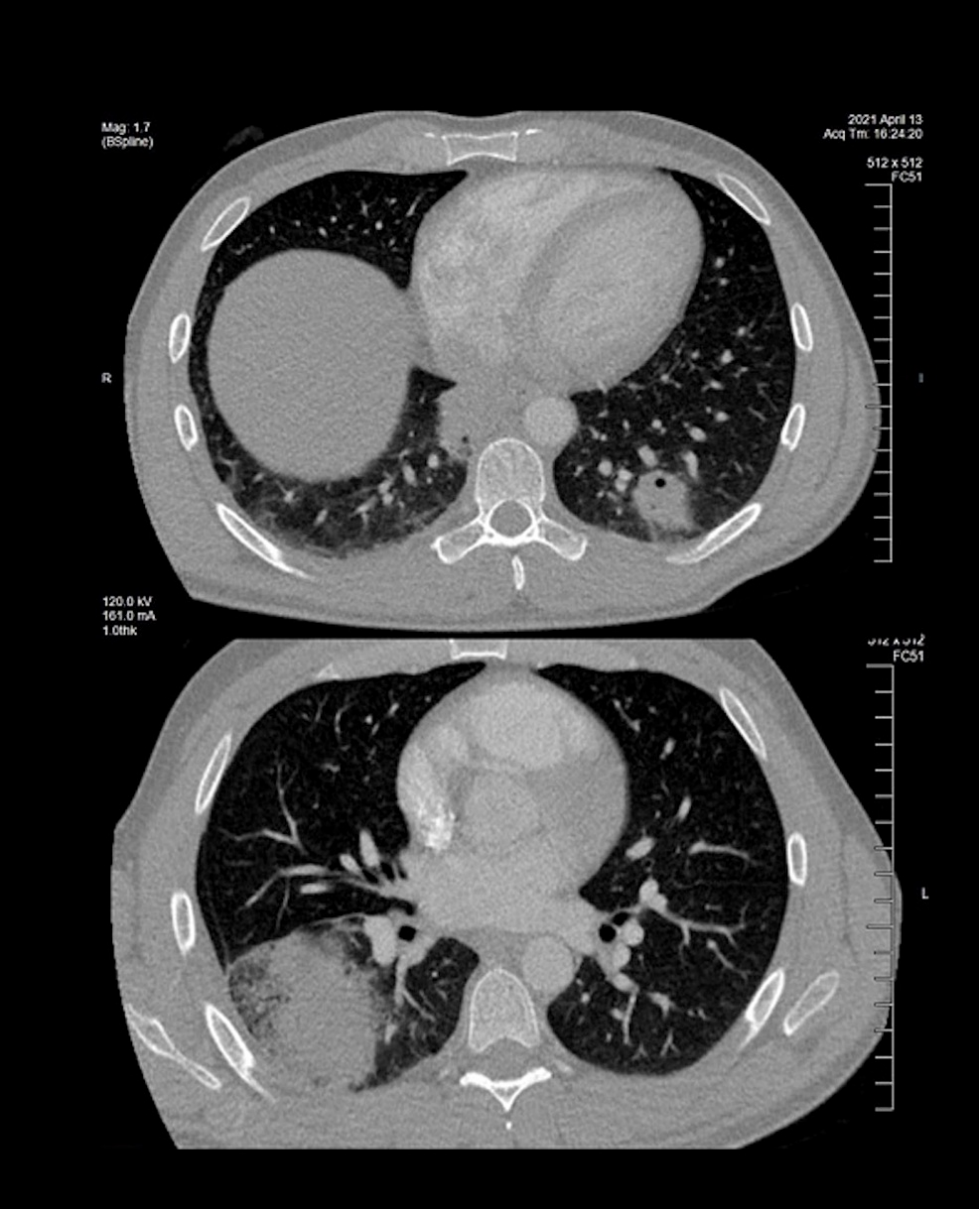
Computed tomographic angiography of the chest (axial view). Axial computed tomographic angiography images of the chest were obtained from the thoracic inlet through the lung bases at 1 mm intervals with IV contrast (75 mL Isovue 370). There were no pulmonary arterial filling defects to suggest acute pulmonary embolus. The bottom image demonstrates a large focus of consolidation and infiltration within the right lower lobe and within the azygous-esophageal recess. The top image shows a 4 cm rounded focal opacity of the left lower lobe with cavitation.

**Figure 3:**
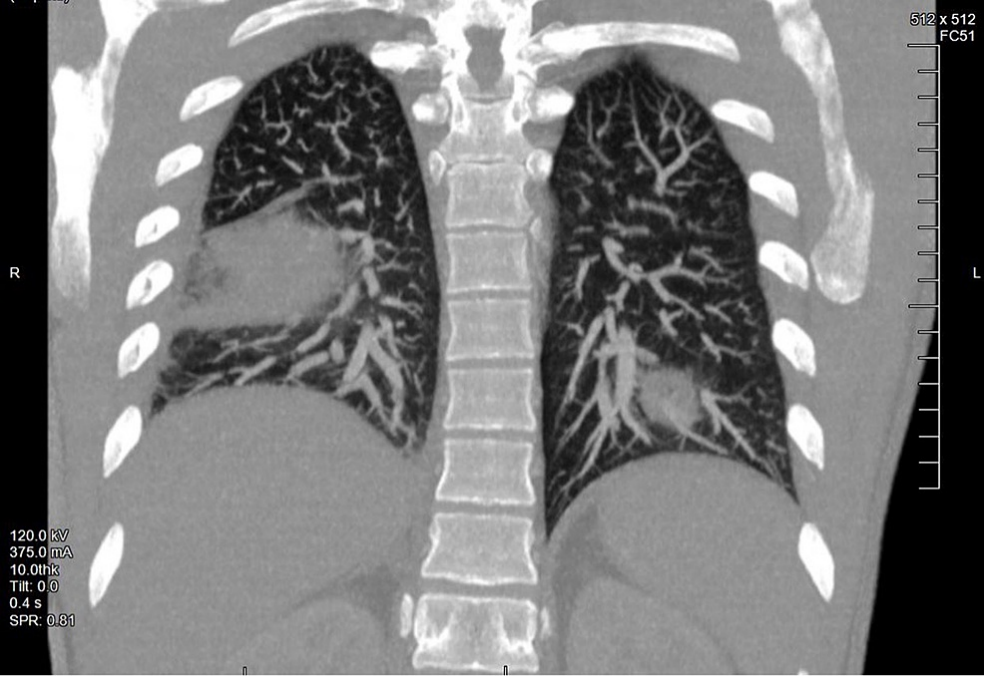
Computed tomographic angiography of the chest (coronal view). Computed tomographic angiography of the chest redemonstrating a large focus of consolidation in the right lower lobe and a 4 cm rounded focal opacity of the left lower lobe with cavitation.

A 31-year-old Caucasian male presented to the ED with a 10-day history of progressively worsening symptoms that started with a sore throat and dysphagia. Five days after the development of initial symptoms, productive cough with orange-colored sputum began. His condition worsened four days before presentation, with the development of fever, chills, night sweats, significant diarrhea, emesis, and pleuritic chest pain. On initial presentation, the patient appeared ill and anxious. He was febrile with a temperature of 102° Fahrenheit, tachycardic with a heart rate of 118 beats per minute, and hypotensive with a blood pressure of 109/54 mmHg. On physical exam, he had a respiratory rate of 18 breaths per minute and oxygen saturation of 95% on room air. His neck was supple with absent lymphadenopathy and both lungs were clear to auscultation. Laboratory work on admission was remarkable for a leukocyte count of 23.2 x 10^9/Liter, a neutrophil count of 85.6% with 16% bands, serum sodium of 132 mmol/Liter, and a D-Dimer of 4.29 mg/L FEU. Initially, one gram of ceftriaxone and 500 mg of azithromycin were administered intravenously in the ED. Tests were then ordered including a monospot and rapid *Streptococcus* A antigen via pharyngeal swab, as well as (1,3)-beta-D-glucan and HIV-1 RNA via serum, and *Legionella pneumophila* urine antigen; all without remarkable findings. A complete viral respiratory panel was negative, and PCR did not detect *Mycoplasma pneumoniae*, SARS-CoV-2, or *Chlamydia pneumoniae*. A portable chest X-Ray performed in the ED demonstrated right upper lobe pneumonia (Figure *1*). A CT angiogram of the chest ordered to rule out pulmonary embolus (Figures 2-3) demonstrated no signs of acute pulmonary emboli. However, findings displayed a large consolidation in the right lower lobe and a 4 cm cavitation in the left lower lobe. Transthoracic ECG was then performed, demonstrating no vegetations or valvular abnormalities. Trans-esophageal ECG was recommended to the patient, but he declined. Next, a CT scan of the neck was ordered, showing no evidence of abnormalities. Without significant improvement in WBC count for three days and persistent fevers >100° Fahrenheit, his antibiotic regimen was escalated to three grams of ampicillin-sulbactam administered intravenously every six hours. This resulted in an improved WBC to 16.1 x 10^9/Liter the following day. The patient had no further fevers and reported improvement in pleuritic chest pain and dysphagia. On day five of hospitalization, the initial blood culture grew a single anaerobic gram-negative bacterium, *F. necrophorum*. The patient continued to show improvement without signs of fever, nausea, or vomiting. A repeat negative blood culture and final WBC count of 14.5 x 10^9/Liter was reported on day eight of hospitalization before the patient was discharged later that afternoon. He was discharged home in a stable condition with 500 mg of amoxicillin-clavulanic acid given orally every twelve hours for seven days and was instructed to follow up with his primary care physician in three weeks for a repeat CT scan of his chest to monitor the resolution of pulmonary lesions. 

## Discussion

*Fusobacterium necrophorum* is a gram-negative, non-spore-forming, anaerobic bacillus. This organism is considered normal human flora in the oropharynx and has been associated with tonsillitis, peritonsillar abscess, and pulmonary lesions with occasional cavitation [2]. The list of etiologic agents for cavitary lesions is extensive. While discovering the particular agent responsible, many clinicians attempt ruling out *Mycobacterium tuberculosis* and malignancy [7]. A rare cause of cavitation is *F. necrophorum*. Most commonly, this bacterium is associated with Lemierre’s syndrome with an estimated incidence of 1/1,000,000 cases worldwide [2]. A frequent site of septic emboli is in the lungs with an incidence of 80% [2]. Radiographic evidence on chest CT typically includes lobar consolidation, pleural effusion, and peripheral pulmonary nodules with occasional cavitation [2]. Studies have also found empyema and lung abscess to be relatively common with additional evidence of both pneumatoceles and pneumothorax [8]. 

After further literature review, few studies have discovered *F. necrophorum* bacteremia with pulmonary emboli exclusive of septic thrombophlebitis [6,9]. While this presentation can progress to post-anginal sepsis, prompt diagnosis and proper treatment are required, entailing high clinical suspicion. Additional studies are warranted to discover the various presentations with this uncommon bacterium.

Initially, empiric antibiotic therapy is administered to target *F. necrophorum* and other oral streptococci [4]. *F. necrophorum* has been found susceptible to penicillin, clindamycin, metronidazole, and chloramphenicol, with variable success in cephalosporins, erythromycin, and tetracyclines [8]. The use of antibiotics resistant to beta-lactamase is the preferred treatment due to cases of failed therapy with penicillin and production of beta-lactamase by *F. necrophorum* [4]. Common empiric treatments include piperacillin-tazobactam 3.375 mg IV every six hours, Imipenem 500 mg IV every six hours, or ceftriaxone 2g IV every 24 hours plus metronidazole 500 mg IV every eight hours [4]. Once culture and sensitivity data are available, antibiotic treatment should be tailored accordingly.

## Conclusions

In closing, we presented a rare case of lung abscess and bacteremia due to *F. necrophorum* without the classic presentation of Lemeirre’s syndrome. Although our patient had positive blood cultures and a cavitary pulmonary lesion, he did not show evidence of internal jugular venous thrombophlebitis. This case was intended to share with the medical community a rare etiologic agent associated with metastatic pulmonary emboli and common treatments recommended, despite uniform guidelines. Patients presenting with progressive pharyngitis, respiratory distress, septic pulmonary emboli, and persistent symptoms despite antibiotics, should have *F. necrophorum* included in the differential diagnosis even in the absence of jugular venous thrombosis.
